# Supplementary material for: Infection fatality rate of SARS-CoV2 in a super-spreading event in Germany
Source: Nat Commun. 2020 Nov 17;11:5829. doi: 10.1038/s41467-020-19509-y (PMC7672059; doi:10.1038/s41467-020-19509-y)
Supplement: Supplementary file 1 — Supplementary Information [file 41467_2020_19509_MOESM1_ESM.pdf]

Supplementary Appendix

Supplement to: Streeck H, Schulte B, Kuemmerer BM, et al. Infection fatality rate of SARS-CoV-2 in a super-spreading event in Germany.

Supplementary Appendix for "Infection fatality rate of SARS-CoV-2 in a super-spreading event in Germany"

Table of contents

		Page
**Figure S1**:	Comparison of the distribution of age groups of the study participants to those in the community of Gangelt, the state NRW and Germany.	3
**Figure S2**:	IgA and IgG levels in study participants of different sex and age.	4
**Figure S3**:	Associations of sex, age and co-morbidities with disease intensity.	5
**Table S1**:	Characteristics of the 88 study participants not evaluable for infection status.	6

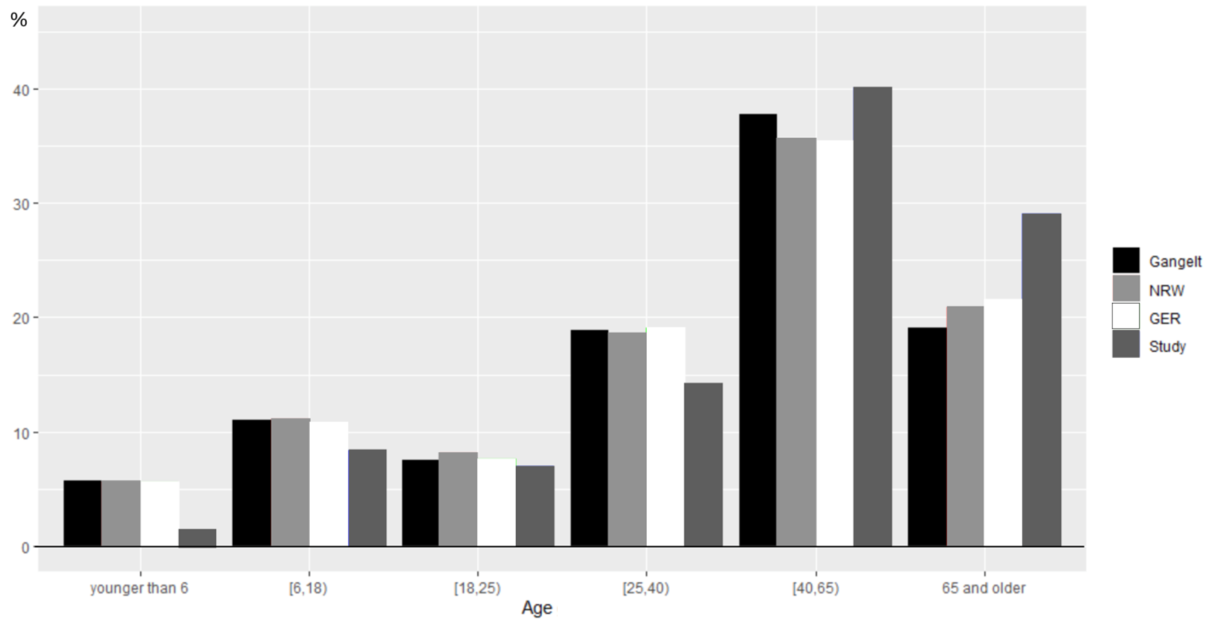

Figure S1: *Comparison of the distribution of age groups of the study participants to those in the community of Gangelt, the state NRW and Germany.* Data were obtained from the Landesdatenbank NRW (reporting date: December 31, 2017) and statista.com (reporting date: December 31, 2018). Note that these sources did not provide data for the age groups used by the RKI. For this reason, the age categories in Tables S1 and S2 differ from the age categories in Figure S1. Source data are provided as a source data file.

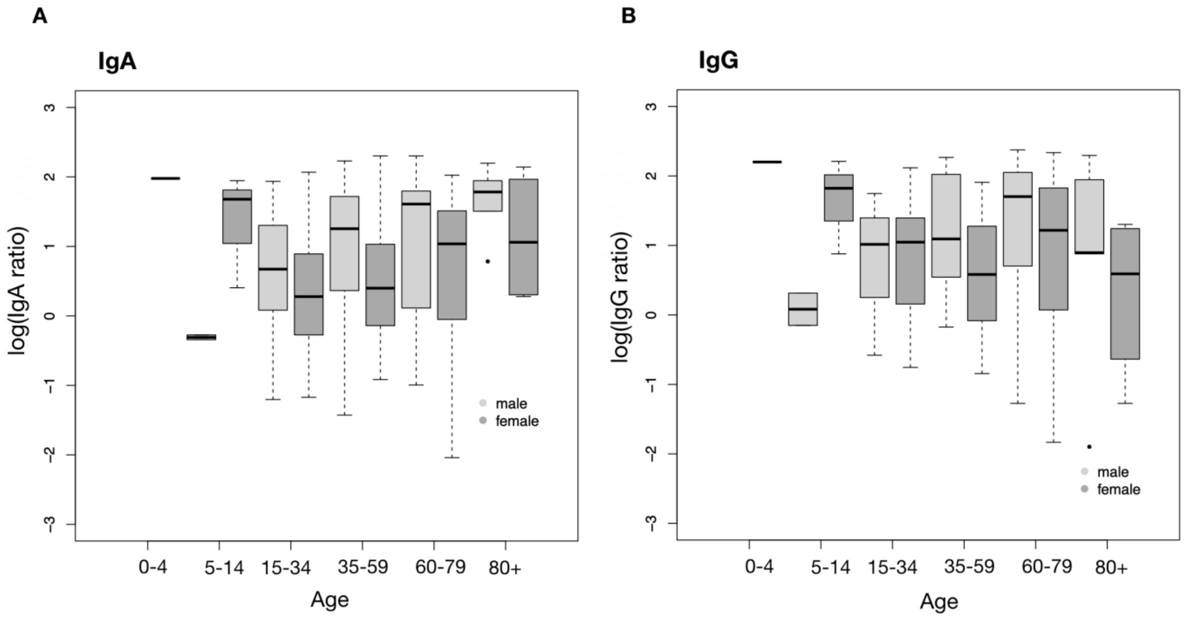

Figure S2: *IgA and IgG levels in study participants of different sex and age.* The boxplots depict the log(IgA) (**A**) and log(IgG) (**B**) levels of the infected study participants grouped by sex and age. No associations were found (i) between log(IgA), log(IgG), and sex in Gaussian models with log(IgA) and log(IgG) as response variables and sex as covariable, and (ii) between log(IgG), sex, and age in a Gaussian model with log(IgG) as response variable and both sex and age as covariables. In a Gaussian model with log(IgA) as response variable and both sex and age as covariables, age (increase = 0.095 [0.018; 0.172] units on log scale per 10 years, p = 0.015) but not sex was found to be associated with log(IgA). Note: Gaussian models were used instead of GEE models because the number of household clusters was large relative to the number of analyzed study participants. Participant numbers are the same as in Figure 6. Source data are provided as a source data file.

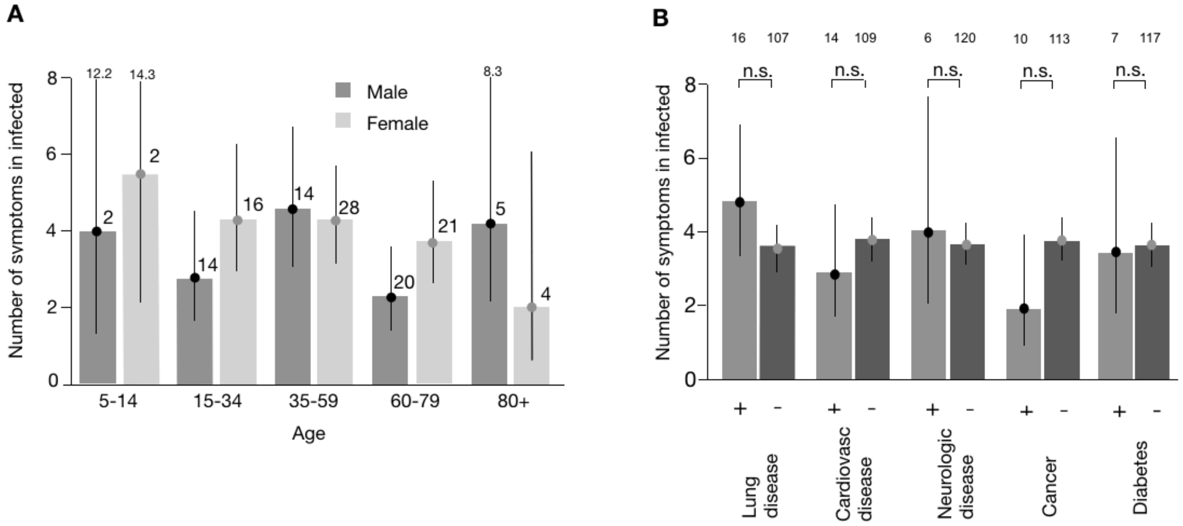

Figure S3: Associations of sex, age and co-morbidities with disease intensity. **A:** Estimated mean numbers of symptoms (filled circles, with 95% CIs) in 55 infected male participants (living in 52 household clusters, dark gray) and 71 infected female participants (living in 66 household clusters, light gray) stratified by age groups. In view of the large number of household clusters relative to the number of persons (52/55, 66/71), estimates were obtained by fitting non-GEE quasi-Poisson models with the number of symptoms as response variable and age as covariable in the male and female subgroups. Bars refer to the empirical mean values. In a quasi-Poisson model with both sex and age as covariables, neither sex (estimated relative mean increase = 1.26 [0.93; 1.71] for females, p = 0.142) nor age (estimated relative mean increase = 0.97 [0.90; 1.04] per 10 years, p = 0.348) were found to be associated with the number of symptoms. Results are based on the 126 infected study participants without missing values in any of the symptom items. Numbers above bars indicate the total number of individuals in the respective group. Note: There were no children aged less than 5 years. Numbers above CIs indicate the upper CI limits. **B:** For each of the co-morbidities, the mean number of symptoms in the infected participants was determined by fitting a non-GEE quasi-Poisson model with the number of symptoms as response variable to the data of the infected study participants (light gray: co-morbidity present (+), dark gray: co-morbidity not present (-)). Quasi-Poisson models were used instead of GEE models for the same reason as in A. Results are based on the 126 infected study participants without missing values in any of the symptom items. Point estimates obtained from the models are represented by filled circles (with 95% CIs). The bars represent the empirical means of the number of symptoms in each of the subgroups. Numbers above bars indicate the total number of individuals in the respective group. No associations between the presence of any of the co-morbidities and the number of symptoms were found (according to Bonferroni-Holm corrected p-values). Analogous results were found when sex and age were added as covariables to the quasi-Poisson models. All statistical tests were two-sided. Adjustments for multiple comparisons were made as indicated. Source data are provided as a source data file.

Table S1. Characteristics of the 88 study participants not evaluable for infection status.

Characteristic	Value
Participants – no. (%) living in household clusters of size	
2	11 (12.50)
3	29 (32.95)
4	28 (31.82)
≥ 5	20 (22.73)
Age	
Median (range)	9.5 yr (1 yr – 84 yr)
Distribution – no. (%)	
<5 yr	28 (31.82)
5-14 yr	28 (31.82)
15-34 yr	12 (13.64)
35-59 yr	10 (11.36)
60-79 yr	9 (10.23)
>79 yr	1 (1.14)
Sex – no. (%)	
Male	36 (41.38)
Female	50 (57.47)
Diverse	1 (1.15)
Missing: 1	
$PCR_{reported}$ – no. (%)	
Yes	13 (14.77)
No	75 (85.23)
$PCR_{reported}$ positive – no. (%)	
Yes	0 (0.00)
No	13 (100.00)
Carnival – no. (%)	
Yes	46 (52.27)
No	42 (47.73)
