# Supplementary material for: Infection fatality rate of SARS-CoV2 in a super-spreading event in Germany
Source: Nat Commun. 2020 Nov 17;11:5829. doi: 10.1038/s41467-020-19509-y (PMC7672059; doi:10.1038/s41467-020-19509-y)
Supplement: Supplementary file 3 — Reporting Summary [file 41467_2020_19509_MOESM3_ESM.pdf]

Corresponding author(s): Hendrik Streeck
Gunther Hartmann

Last updated by author(s): Oct 3, 2020

Reporting Summary

Nature Research wishes to improve the reproducibility of the work that we publish. This form provides structure for consistency and transparency in reporting. For further information on Nature Research policies, see our Editorial Policies and the Editorial Policy Checklist.

Please do not complete any field with "not applicable" or n/a. Refer to the help text for what text to use if an item is not relevant to your study.
For final submission: please carefully check your responses for accuracy; you will not be able to make changes later.

Statistics

For all statistical analyses, confirm that the following items are present in the figure legend, table legend, main text, or Methods section.

n/a	Confirmed	
☐	☒	The exact sample size (*n*) for each experimental group/condition, given as a discrete number and unit of measurement
☐	☒	A statement on whether measurements were taken from distinct samples or whether the same sample was measured repeatedly
☐	☒	The statistical test(s) used AND whether they are one- or two-sided<br>*Only common tests should be described solely by name; describe more complex techniques in the Methods section.*
☐	☒	A description of all covariates tested
☐	☒	A description of any assumptions or corrections, such as tests of normality and adjustment for multiple comparisons
☐	☒	A full description of the statistical parameters including central tendency (e.g. means) or other basic estimates (e.g. regression coefficient) AND variation (e.g. standard deviation) or associated estimates of uncertainty (e.g. confidence intervals)
☐	☒	For null hypothesis testing, the test statistic (e.g. F, t, r) with confidence intervals, effect sizes, degrees of freedom and P value noted<br>*Give P values as exact values whenever suitable.*
☐	☒	For Bayesian analysis, information on the choice of priors and Markov chain Monte Carlo settings
☐	☒	For hierarchical and complex designs, identification of the appropriate level for tests and full reporting of outcomes
☐	☒	Estimates of effect sizes (e.g. Cohen's *d*, Pearson's *r*), indicating how they were calculated

Our web collection on statistics for biologists contains articles on many of the points above.

Software and code

Policy information about availability of computer code

Data collection	RedCap version 9.5.6
Data analysis	Statistical analysis was carried out using version 4.0.0 of the R Language for Statistical Computing(R Core Team 2020: R: A Language and Environment for Statistical Computing. R Foundation for Statistical Computing, Vienna, Austria) and version 9.4 of the SAS System for Windows (copyright © 2002-2012 by SAS Institute Inc., Cary, NC, USA).

For manuscripts utilizing custom algorithms or software that are central to the research but not yet described in published literature, software must be made available to editors and reviewers. We strongly encourage code deposition in a community repository (e.g. GitHub). See the Nature Research guidelines for submitting code & software for further information.

Data

Policy information about availability of data

All manuscripts must include a data availability statement. This statement should provide the following information, where applicable:

- Accession codes, unique identifiers, or web links for publicly available datasets
- A list of figures that have associated raw data
- A description of any restrictions on data availability

The data contain information that could compromise the privacy of research participants. Data sharing restrictions imposed by national and trans-national data sharing laws prohibit general sharing of data. However, upon submission of a proposal to the principal investigator of this study and approval of this proposal by i) the principal investigator, ii) the Ethics Committee of the University of Bonn and iii) the data protection officer of the University Hospital Bonn, data collected for the study can be made available to other researchers.

Field-specific reporting

Please select the one below that is the best fit for your research. If you are not sure, read the appropriate sections before making your selection.

- [x] Life sciences
- [ ] Behavioural & social sciences
- [ ] Ecological, evolutionary & environmental sciences

Life sciences study design

All studies must disclose on these points even when the disclosure is negative.

Sample size	In the absence of any pilot data on SARS-CoV-2 infection rate in Gangelt, sample size calculations were based on the WHO population-based age-stratified seroepidemiological investigation protocol for COVID-19 virus infection. According to the recommendations stated in the protocol, a size of 200 samples is sufficient to estimate SARS-CoV-2-prevalence rates <10% with an expected margin of error (defined by the expected width of the 95% confidence interval associated with the seroprevalence point estimate obtained using binomial likelihood) smaller than 10%. In order to rule out larger margins of error due to dependencies of persons living in the same household and to be able to analyze seroprevalence (i.e., infection rates) also in subgroups defined by participant age, it was planned to recruit 1,000 participants living in at least 300 households.
Data exclusions	Data exclusion criteria are described in Fig. 1B. Furthermore, the exclusion of data is indicated and explained in the main text wherever applicable.
Replication	Indicated wherever applicable.
Randomization	Not applicable since this is an observational study. Covariates were controlled by fitting regression models (in particular, GEE models).
Blinding	Not applicable since this is an observational study.

Reporting for specific materials, systems and methods

We require information from authors about some types of materials, experimental systems and methods used in many studies. Here, indicate whether each material, system or method listed is relevant to your study. If you are not sure if a list item applies to your research, read the appropriate section before selecting a response.

Materials & experimental systems

n/a	Involved in the study	
☐	☒	Antibodies
☐	☒	Eukaryotic cell lines
☒	☐	Palaeontology and archaeology
☒	☐	Animals and other organisms
☐	☒	Human research participants
☒	☐	Clinical data
☒	☐	Dual use research of concern

Methods

n/a	Involved in the study	
☒	☐	ChIP-seq
☒	☐	Flow cytometry
☒	☐	MRI-based neuroimaging

Antibodies

Antibodies used	We used commercially available ELISA kits that measure anti-SARS-CoV-2 IgA (Euroimmun, EI 2606-9620 A) and anti-SARS-CoV-2 IgG (EI 2606-9620 G).
Validation	The ELISAs were validated by the manufacturer Euroimmun.

Eukaryotic cell lines

Policy information about cell lines

Cell line source(s)	Vero E6 cells kindly provided by Ralf Bartenschlager, Heidelberg University
Authentication	cell line used was not authenticated
Mycoplasma contamination	tested negative for mycoplasma contamination in Bonn
Commonly misidentified lines (See ICLAC register)	not applicable

Human research participants

Policy information about studies involving human research participants

Population characteristics	Please see Table 1.
Recruitment	Please see Methods section.
Ethics oversight	The study was approved by the Ethics Committee of the Medical Faculty of the University of Bonn (approval number 085/20).

Note that full information on the approval of the study protocol must also be provided in the manuscript.

This checklist template is licensed under a Creative Commons Attribution 4.0 International License, which permits use, sharing, adaptation, distribution and reproduction in any medium or format, as long as you give appropriate credit to the original author(s) and the source, provide a link to the Creative Commons license, and indicate if changes were made. The images or other third party material in this article are included in the article's Creative Commons license, unless indicated otherwise in a credit line to the material. If material is not included in the article's Creative Commons license and your intended use is not permitted by statutory regulation or exceeds the permitted use, you will need to obtain permission directly from the copyright holder. To view a copy of this license, visit http://creativecommons.org/licenses/by/4.0/
